# Autophagy activation and SREBP‐1 induction contribute to fatty acid metabolic reprogramming by leptin in breast cancer cells

**DOI:** 10.1002/1878-0261.12860

**Published:** 2020-12-05

**Authors:** Duc‐Vinh Pham, Nirmala Tilija Pun, Pil‐Hoon Park

**Affiliations:** ^1^ College of Pharmacy Yeungnam University Gyeongsan Korea; ^2^ Research Institute of Cell Culture Yeungnam University Gyeongsan Korea

**Keywords:** autophagy, breast cancer, leptin, metabolic reprogramming, SREBP‐1

## Abstract

Leptin, a hormone predominantly derived from adipose tissue, is well known to induce growth of breast cancer cells. However, its underlying mechanisms remain unclear. In this study, we examined the role of reprogramming of lipid metabolism and autophagy in leptin‐induced growth of breast cancer cells. Herein, leptin induced significant increase in fatty acid oxidation‐dependent ATP production in estrogen receptor‐positive breast cancer cells. Furthermore, leptin induced both free fatty acid release and intracellular lipid accumulation, indicating a multifaceted effect of leptin in fatty acid metabolism. These findings were further validated in an MCF‐7 tumor xenograft mouse model. Importantly, all the aforementioned metabolic effects of leptin were mediated via autophagy activation. In addition, SREBP‐1 induction driven by autophagy and fatty acid synthase induction, which is mediated by SREBP‐1, plays crucial roles in leptin‐stimulated metabolic reprogramming and are required for growth of breast cancer cell, suggesting a pivotal contribution of fatty acid metabolic reprogramming to tumor growth by leptin. Taken together, these results highlighted a crucial role of autophagy in leptin‐induced cancer cell‐specific metabolism, which is mediated, at least in part, via SREBP‐1 induction.

## Graphical abstract

Leptin has been shown to promote breast tumor growth via autophagy induction. Here, we demonstrate that SREBP‐1 induction downstream of leptin‐dependent autophagy activation triggers a profound reprogramming in fatty acid metabolism. This metabolic reprogramming increases ATP production and fuels breast cancer‐cell growth. Our study suggests autophagy and SREBP‐1 as potential therapeutic targets in obesity‐linked breast cancer.

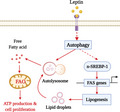

**Abbreviations**
2‐DG: 2‐deoxyglucose
3‐MA: 3‐methyladenine
ACC‐1: acetyl‐CoA carboxylase 1
ACLY: ATP citrate lyase
ER: estrogen receptor
FADS1: fatty acid desaturase 1
FADS2: fatty acid desaturase 2
FAO: fatty acid oxidation
FAS: fatty acid synthesis
FASN: fatty acid synthase
FFA: free fatty acid
IHC: immunohistochemistry
SCD‐1: stearoyl‐CoA desaturase‐1
SREBP‐1: sterol regulatory element‐binding protein 1

## 1 Introduction

Leptin, an adipokine primarily derived from adipocytes, modulates diverse biological responses. In addition to its critical roles in the regulation of appetite and energy balance [1], a large body of evidence has indicated that leptin induces initial development and progression of various types of cancers, in particular obesity‐associated cancers, such as hepatic, colon, and breast cancer [2]. Leptin exhibits potent oncogenic actions and acts on different stages of cancer, including cell proliferation, angiogenesis, metastasis, and drug resistance, via multiple mechanisms such as activation of MEK/ERK1/2 and PI3K/Akt signaling pathways, autophagy induction, and NLRP3 inflammasome activation [3, 4, 5, 6].

Metabolic reprogramming, a phenomenon by which cancer cells can satisfy the energy requirement for their survival and proliferation in a harsh tumor microenvironment, has long been considered a crucial hallmark of cancers [7]. In addition to alterations in glucose metabolism, commonly called the Warburg effect, metabolic rewiring of cancer cells has been recently described for a wide range of other metabolic pathways, including amino acid/lipid metabolism, mitochondrial biogenesis, pentose phosphate pathway, and macromolecule biosynthesis [8, 9, 10, 11]. Among them, alterations in lipid metabolic pathway, specifically fatty acid synthesis (FAS) and fatty acid oxidation (FAO), have received an increasing attention in recent years due to their critical roles in tumor initiation, development, progression, and metastasis [12, 13, 14, 15, 16]. Accumulating evidence indicates that cancer cells usually show a unique behavior in terms of lipid metabolism compared to normal cells. For instance, while most adult mammalian cells acquire fatty acid (FA) from the bloodstream, tumor cells are frequently associated with increased *de novo* biosynthesis of FA, a process catalyzed by concerted actions of various enzymes, most of which are regulated by sterol regulatory element‐binding protein 1 (SREBP‐1) [15, 17, 18]. Interestingly, an increase in lipid accumulation in cancer cells can also be coupled with lipolysis [15, 19, 20], although these processes rarely co‐exist in normal metabolic conditions. Like many other tumors, breast tumors show a lipogenic phenotype with profound changes in acquisition, synthesis, and utilization of fatty acid [21]. However, little is known about what factors determine the extensive metabolic reprogramming in breast cancer cells.

Autophagy, an intracellular self‐digestive process for removing damaged cytoplasmic constituents, has been shown to play a role in modulation of cancer cell survival and proliferation [22]. Recently, there has been an increasing appreciation of the contribution of this complex process to cancer cell‐specific metabolic changes [23]. In certain tumor types, autophagy may promote tumor growth by providing tumor cells with diverse metabolic fuel sources such as glucose, amino acids, nucleosides, and fatty acid. However, the direct involvement of autophagy in lipid metabolism was under‐evaluated until a novel function of autophagy, termed as lipophagy, was discovered [24]. Accordingly, autophagy is essentially required for lipid droplet breakdown and thus plays a key role in release of free fatty acid (FFA) from storage sites. Hence, it is not surprising that inhibition of autophagy flux impedes lipid metabolism in cancer cells, which in turn leads to a reduction in energy production and cell proliferation [25, 26]. On the contrary, other studies have also reported that autophagy is implicated in lipid synthesis and accumulation [27, 28], suggesting a complicated and context‐dependent role of autophagy in cancer cell‐specific lipid metabolism.

Although leptin has been well known to support tumor growth through multiple mechanisms [3, 6, 29], little effort has been made to examine the various metabolic effects in cancer cells and its potential roles in the pro‐carcinogenic effects of this adipokine. Limited data suggest that leptin induces a substantial metabolic reprogramming in malignant cells through an improvement in mitochondrial function and cellular respiration [30], elevation of ATP production [31], and increase in use of fatty acid for energy generation by FAO stimulation [31, 32]. However, molecular mechanisms underlying these changes and the linkage between altered lipid metabolism and tumorigenesis by leptin have not been completely elucidated. Likewise, the mechanisms by which autophagy drives tumor growth remain elusive.

To bridge the knowledge gap between leptin‐mediated growth of breast cancer cell and lipid metabolic reprogramming, the present study was conducted to unveil the effects of leptin on production, accumulation, and utilization of fatty acid, as well as their involvement in the growth of breast cancer cells. Herein, we demonstrate that leptin increases intracellular lipid deposits through SREBP‐1‐mediated stimulation of *de novo* biosynthesis of fatty acid. Furthermore, leptin upregulates intracellular FFA levels and stimulates the use of fatty acid for ATP production via FAO induction in estrogen receptor (ER)‐positive breast cancer cells. These changes in lipid metabolism are essentially required for leptin‐promoted tumor growth. Finally, we have also demonstrated that all these effects of leptin on lipid metabolism and growth of breast cancer cell are driven by autophagy activation and SREBP‐1 induction.

## 2 Materials and methods

### 2.1 Materials

All the cell culture reagents were provided by HyClone Laboratories (South Logan, UT, USA). Recombinant mouse leptin, Bafilomycin A1, and fatostatin were purchased from Sigma‐Aldrich (St. Louis, MO, USA). Oligomycin and Etomoxir were obtained from Cayman Chemical (Ann Arbor, MI, USA). Bodipy 493/503 was purchased from Invitrogen (Carlsbad, CA, USA). CellTiter 96^®^ AQueous One Solution Cell Proliferation Assay (MTS) was procured from Promega Corporation (Madison, WI, USA). Cycle test plus DNA reagent kit was provided by BD Biosciences (San Jose, CA, USA). Primary antibodies against phospho‐Akt, total Akt, LC3B, p27 Kip1, and FASN were purchased from Cell Signaling Technology Inc. (Beverly, MA, USA). Anti‐cyclin D1 antibody was obtained from Santa Cruz Biotechnology (Dallas, TX, USA). The antibodies against β‐actin and SREBP‐1 were procured from Thermo Scientific (Waltham, MA, USA) and Abcam (Cambridge, MA, USA), respectively. The anti‐rabbit and anti‐mouse secondary antibodies conjugated with horseradish peroxidase (HRP) were provided by Thermo Scientific. The biotinylated anti‐rabbit secondary antibody was acquired from Vector Laboratories Inc (Burlingame, CA, USA). All other chemicals and reagents were purchased from Sigma‐Aldrich unless mentioned elsewhere.

### 2.2 Cell culture

MCF‐7, T47‐D, and MDA‐MB‐231 cell lines were purchased from American Type Culture Collection (ATCC, Rockville, MD, USA) and maintained in Dulbecco's modified Eagle's medium (DMEM) (MCF‐7 cells) or RPMI 1640 (T47D and MDA‐MB‐231 cells) containing 10% FBS and 1% penicillin–streptomycin. Cells were routinely cultured in an incubator at 37 °C under a humidified atmosphere of 95% O_2_ and 5% CO_2_.

### 2.3 Measurement of total cellular ATP

Cellular ATP levels were measured using Luminescent ATP Detection Assay Kit (Abcam) according to manufacturer's instructions. Briefly, cells were seeded in 96‐well white plates at the density of 2 × 10^4^ cells per well. After overnight incubation, cells were treated with leptin and an appropriate agent as indicated. Cells were then lysed by incubation with detergent for 5 min to release and stabilize ATP. ATP levels were determined by measurement of the luminescence formed by oxidation of the substrate d‐luciferin in presence of luciferase using a microplate reader (Fluostar Optima, BMG Labtech Inc, Ortenberg, Germany).

### 2.4 Determination of cellular acetyl‐CoA levels

Acetyl‐CoA levels were assessed by PicoProbe™Acetyl‐CoA Fluorometric Assay Kit (BioVision, Milpitas, CA, USA) according to manufacturer's protocol. After treatments as indicated, 10^6^ cells were collected and lysed in the assay buffer. Cell lysates were deproteinized using perchloric acid, followed by incubation with CoA Quencher for removing free CoASH, malonyl CoA and succ‐CoA. Finally, the reaction mix containing substrates, conversion enzyme, enzyme mix, PicoProbe reagent, and sample aliquots was incubated at 37 °C for 10 min before fluorescence was acquired at 540/595 nm.

### 2.5 Fatty acid oxidation assay

FAO activity was determined by Fatty Acid Oxidation Complete Assay Kit (Abcam) in line with manufacturer's recommendations. Briefly, cells were seeded in 96‐well clear bottom black walled plates at the density of 2 × 10^4^ cells per well. After overnight incubation, cells were treated with leptin in the absence or presence of inhibitors as indicated. Cells were then washed with FA‐free media (containing base measurement media supplemented with 0.5 mm l‐carnitine and 2.5 mm glucose) followed by adding FA measurement media (containing 150 µm FAO conjugate). Extracellular oxygen consumption reagent was then added before wells were sealed by two drops of high sensitivity mineral oil. Finally, fluorescence was immediately monitored at Ex/Em of 360/670 using a microplate reader (Spark™ 10M multimode microplate reader; Tecan, Mannedorf, Switzerland).

### 2.6 Measurement of neutral lipid accumulation

Neutral lipid content was determined as previously described [33]. Cells were seeded at the density of 5 × 10^5^ cells per 35‐mm dish. After treatment with leptin and inhibitors as indicated, cells were incubated with Bodipy 493/503 staining solution (2 µm) in dark at 37 °C for 20 min before a single cell suspension was collected by trypsinization. Cells were then subjected to flow cytometry analysis using BD FACSCalibur™ (BD Biosciences, San Jose, CA, USA), and mean fluorescence intensity values were obtained by flowjo 7.6 Software (FlowJo LLC, Ashland, OR, USA).

For visualization of intracellular lipid droplets, cells were seeded in 8‐well glass slides at the density of 5 × 10^4^ cells/well. After Bodipy staining as described above, cells were fixed with 4% formaldehyde and counterstained with DAPI. The images of lipid droplets were finally captured using a confocal microscopy (Nikon, Tokyo, Japan).

### 2.7 Free fatty acid assay

The levels of long‐chain FFAs were determined using Free Fatty Acid Assay Kit (Abcam) according to manufacturer's protocol. Briefly, cells were seeded in 35‐mm dishes at the density of 5 × 10^5^ cells/dish. After treatment with leptin and inhibitors as indicated, cells were collected by scraping and equal number of cells were homogenized in chloroform solution containing Triton X‐100. After centrifuge, organic phase was separated, and lipid contents were obtained and solubilized in assay buffer. For fatty acid detection, dried lipid samples were first incubated with acyl‐CoA synthetase reagent in a clear bottom black walled plate for 30 min at 37 °C to convert fatty acid to their CoA derivatives. A reaction mixture (containing Fatty acid probe, Enzyme mix and Enhancer reagent) was then added into each and further incubated for 30 min at 37 °C. FFA levels were finally determined by measuring fluorescence at Ex/Em = 535/587 nm.

### 2.8 Cell viability assay

Cell viability was determined by MTS assay as described previously [34]. Briefly, cells were seeded in 96‐well plates at the density of 2 × 10^4^ cells/well. After overnight incubation, cells were treated with leptin in the absence or presence of inhibitors for 48 h followed by a further incubation with 3‐(4, 5‐dimethylthiazol‐2‐yl)‐5‐(3‐carboxymethoxyphenyl)‐2‐(4‐sulfopheny)‐2H‐tetrazolium (MTS, 20 µL) reagent for 2 h at 37 °C. The number of viable cells was examined by measuring the absorbance of resultant formazan dye at 490 nm using the SPECTROstar^Nano^ microplate reader (BMG Labtech Inc).

### 2.9 SREBP‐1 DNA binding activity assay

Specific DNA binding activity of SREBP‐1 was measured using SREBP‐1 Transcription Factor Assay Kit (Cayman Chemical, Ann Arbor, MI, USA) based on the manufacturer's instructions. Cells were seeded in 60‐mm dishes at the density of 1 × 10^6^ cells/dish. After treatments as indicated, nuclear extracts were prepared using nuclear extraction buffer (250 mm sucrose, 20 mm Hepes, 10 mm KCl, 1.5 mm MgCl_2_, 1 mm EDTA, 1 mm EGTA, 1 mm DTT, supplemented with protease and phosphatase inhibitor cocktail). For detection of SREBP‐1 specific dsDNA biding activity, nuclear protein (20 µg) was added into wells coated with a consensus dsDNA sequence containing the SREBP response element followed by overnight incubation at 4 °C. The dsDNA‐SREBP‐1 complex was then detected by sequential incubation with an anti‐SREBP‐1 primary antibody, a compatible HRP‐conjugated secondary antibody, and substrate solution. Finally, the absorbance was measured at 450 nm.

### 2.10 RNA isolation, reverse transcription (RT), and quantitative PCR (qPCR)

For the measurement of mRNA levels of the genes of interest, total RNAs were extracted and isolated using Qiagen lysis reagent (Qiagen, Germantown, MD, USA) according to the manufacturer's instructions. Complementary DNA was subsequently synthesized from one microgram of total RNA using Go Script reverse transcription system (Promega Corporation). Quantitative real‐time PCR amplification (qPCR) was then performed with a Roche LightCycler 2.0 (Mannheim, Germany) using the absolute qPCR SYBR green capillary mix AB gene system (Thermo Scientific, Loughborough, UK) at 95 °C for 15 min followed by 40 cycles at 95 °C for 15 s, 60 °C for 30 s and 72 °C for 30 s. The primer sequences used for amplification of target genes are shown in Table S1.

### 2.11 Preparation of cellular extracts and western blot analysis

Cells or tumor tissues were lysed in radio‐immunoprecipitation assay (RIPA) lysis buffer containing a halt protease and phosphatase inhibitor cocktail. Total lysates were then centrifuged to remove debris and protein concentration was measured by Pierce™ BCA Protein Assay Kit (Thermo Scientific). Equal amounts (30–50 µg) of lysates were separated in sodium dodecyl sulfate polyacrylamide gel (7.5–15%) by electrophoresis, transferred to PVDF membranes, and blocked with 5% skim milk for reducing nonspecific binding. Membranes were then incubated with the primary antibody overnight at 4 °C followed by further incubation with an appropriate HRP‐conjugated secondary antibody for 1 h at room temperature. An enhanced chemiluminescence (ECL) detection system was used to detect immunocomplexes and images were acquired using the Fujifilm LAS‐4000 mini (Fujifilm, Tokyo, Japan).

### 2.12 Transient transfection with small interfering RNA

Cells were transfected with small interfering RNA (siRNA) targeting the specific gene or scrambled control siRNA using HiPerFect Transfection Reagent (Qiagen, Hilden, Germany) according to the manufacturer's guidelines. The gene silencing efficiency was monitored by western blot analysis after 48 h of transfection. The siRNA duplexes used in this study were synthesized by Bioneer (Daejeon, South Korea) with sequences as shown in Table S1.

### 2.13 Cell cycle analysis

The cell cycle analysis was carried out using BD Cycletest™ Plus DNA kit (BD Biosciences) as described previously [35]. Briefly, cells were seeded in 35‐mm dishes at the density of 2 × 10^4^ cells/dish. After overnight incubation, cells were treated with leptin and inhibitors as indicated and maintained in a serum reduced medium (containing 0.1% FBS) for 48 h. For determination of cell cycle phase distribution, cells were first collected in citrate buffer by scraping followed by sequentially incubation with Solution A (containing trypsin in a spermine tetrahydrochloride detergent buffer for digestion of cell membranes and cytoskeletons), Solution B (containing trypsin inhibitor and ribonuclease A to inhibit the trypsin activity and to digest the RNA), and Solution C (containing propidium iodide for DNA staining). Finally, the samples were acquired on a flow cytometer (BD FACSverse, BD Biosciences, San Jose, CA, USA) and the proportion of the cells in each cell cycle phase was calculated using flowjo 7.6 Software.

### 2.14 Preparation of xenograft model

All the animal studies were performed in compliance with the guidelines of Yeungnam University Institutional Animal Care and Use Committee (IACUC). The experimental protocols were reviewed and approved by Yeungnam University (Protocol number: YU‐2019‐021). For generation of tumor xenografts, 10^7^ matrigel‐mixed MCF‐7 cells were injected into the rear flanks of 4‐week‐old male BALB/c nude mice (Orient Ltd., Osan, South Korea). After the tumors reached about 100 mm^3^, mice were randomly divided into four groups (*n* = 5) and exposed to one of the following treatments: leptin (1 mg·kg^−1^) alone, leptin combination with 3‐MA (1 mg·kg^−1^), 3‐MA alone (1 mg·kg^−1^), or phosphate‐buffered saline (served as control group). All treatments were given by intraperitoneal injection every 36 h for 4 weeks. Tumor volume was monitored twice a week during the treatment period using digital Vernier caliper and tumor size (*V*) was calculated as, *V* = (width)^2^ × length/2. Finally, tumor samples were excised followed by measurement, weighing, and storage for further analysis by western blot analysis and IHC.

### 2.15 Immunohistochemical analysis (IHC)

Tumor tissues were fixed with 4% paraformaldehyde for 36 h, and tumor sections (30 µm) were prepared using a freezing sliding microtome (Microm HM 450; Thermo Scientific). The sections were then incubated with 3% hydrogen peroxide for 20 min for removing endogenous peroxidase activity, followed by blocking with 5% goat serum. A primary antibody and a respective biotinylated secondary antibody were consecutively applied to the sections before immunocomplexes were detected by sequential incubation with avidin‐biotin‐peroxidase solution (Vector Laboratories Inc.) and diaminobenzidine (Sigma‐Aldrich). Images were acquired using a light microscope (BX41 TF; Olympus, Tokyo, Japan).

### 2.16 Statistical analysis

Data were expressed as mean ± SEM from at least three independent experiments. Statistical analysis was performed by graphpad prism software version 5.01 (San Diego, CA, USA). Significant differences were estimated using the one‐way analysis of variance (ANOVA), followed by the Tukey's *post hoc* multiple comparison tests. *P* values of lower than 0.05 were considered to be statistically significant.

## 3 Results

### 3.1 Leptin induces fatty acid oxidation‐dependent ATP production in breast cancer cells

**Fig. 1:**
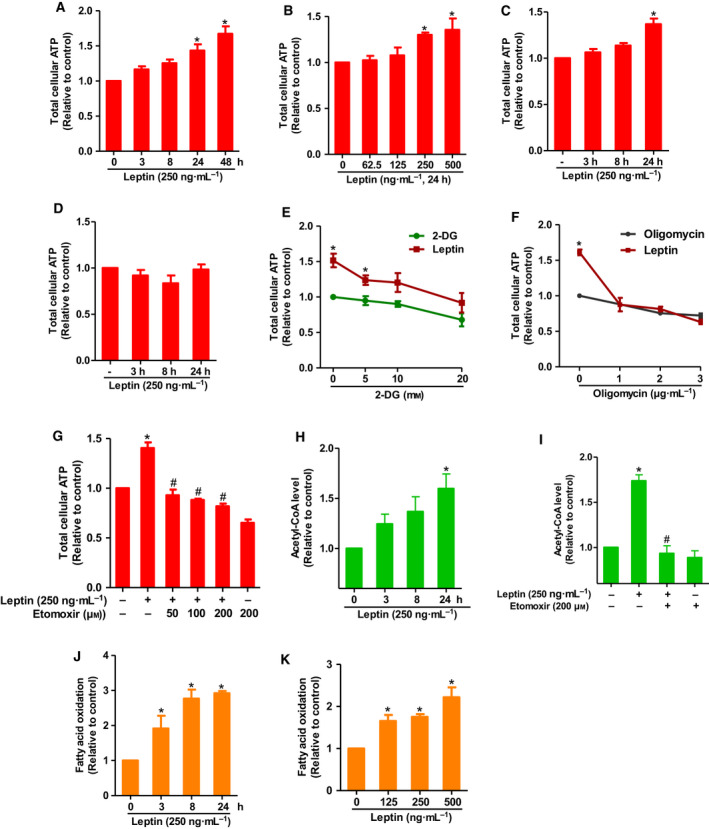
Effects of leptin on cellular ATP level and FAO in breast cancer cells. (A, B) MCF‐7 cells were treated with leptin (250 ng·mL^−1^) for the indicated time duration (A) or different concentrations of leptin for 24 h (B). Total cellular ATP level was measured as described in Materials and methods section. (C, D) T47D cells (C) or MDA‐MB‐231 (D) cells were treated with leptin (250 ng·mL^−1^) for the indicated time duration and ATP level was examined. (E–G) MCF‐7 cells were treated with leptin (250 ng·mL^−1^) in combination with different concentrations of 2‐DG (E), Oligomycin (F), or Etomoxir (G) in DMEM containing high glucose (25 mm). In these experiments, cells were incubated with leptin for 24 h followed by further incubation with 2‐DG, Oligomycin, and Etomoxir for 30, 40, and 60 min, respectively, before ATP levels were measured. (H, I) MCF‐7 cells were treated with leptin (250 ng·mL^−1^) for the indicated time duration (H) or leptin (250 ng·mL^−1^) for 24 h in combination with Etomoxir (200 µm) (I). Acetyl‐CoA levels were determined. (J, K) MCF‐7 cells were treated with leptin (250 ng·mL^−1^) for different time durations (J) or indicated concentrations of leptin for 24 h (K). Fatty acid consumption rate was determined by FAO assay as described in Materials and methods section. The values represent the fold change in comparison to the control cells and are expressed as mean ± SEM, *n* = 3. *P < 0.05 compared to control cells; ^#^ *P* < 0.05 compared to the cells treated with leptin alone.

Adenosine triphosphate (ATP) is a key molecule in living cells. In tumor tissues, higher levels of ATP are generated to meet the energy demand of fast‐growing cancer cells. Thus, to investigate whether leptin affects cancer cell‐specific metabolism, we first examined the effect of leptin on ATP production in breast cancer cells. Leptin increased ATP production in a time‐ and dose‐dependent manner in MCF‐7 breast cancer cells (Fig. 1A,B). Interestingly, similar effects of leptin on cellular ATP production were also observed in T47D cells, another estrogen receptor (ER)‐positive breast cancer line (Fig. 1C), but not in MDA‐MB‐231, triple‐negative breast cancer cells (Fig. 1D), suggesting a critical role of ER signaling in leptin‐stimulated ATP production. Given that ATP is generated in mammary cells mainly via two sequential pathways: glycolysis and mitochondrial oxidative phosphorylation, we next examined individual contribution of these pathways to ATP production induced by leptin. As shown in Fig. 1E,F, both 2‐Deoxy glucose (2‐DG), a glycolytic inhibitor, and Oligomycin, an inhibitor of ATP synthase, decreased leptin‐induced ATP production. Herein, while cotreatment with Oligomycin decreased cellular ATP level almost to that of the control (Fig. 1F), ATP generation promoted by leptin was only partially reversed by 2‐DG (Fig. 1E). We next asked if fatty acid can be used as a fuel source for leptin‐stimulated ATP production in breast cancer cells. To verify this possibility, cells were cotreated with leptin and Etomoxir, which is a pharmacological inhibitor of carnitine palmitoyltransferase I (CPT‐1) and by which acts as a FAO inhibitor. As expected, leptin‐induced ATP production was significantly inhibited by cotreatment with Etomoxir (Fig. 1G), suggesting that leptin uses fatty acid (FFA) for ATP production in breast cancer cells. Taking into consideration the possible effects of treatments on cell viability, which in turn may produce changes in the cellular ATP level, we examined effects of 2‐DG, oligomycin, and etomoxir alone or in combination with leptin on the number of MCF‐7 cells, and found that there was no significant effect of these treatments on MCF‐7 cell viability (data not shown). During generation of ATP molecules from fatty acids, acyl‐CoAs are oxidized in mitochondria to form acetyl‐CoA. Therefore, to confirm that leptin promotes the use of fatty acids for FAO, we next examined the effect of leptin on the cellular acetyl‐CoA levels and found that leptin increased acetyl‐CoA levels in MCF‐7 cells (Fig. 1H). Importantly, this effect was abolished by Etomoxir (Fig. 1I), implying that enhanced acetyl‐CoA production in response to leptin stimulation is derived from FAO. Finally, the involvement of fatty acid metabolism in leptin‐induced energy production was further confirmed by FAO activity assay whereby leptin was found to enhance FAO in a time‐ and dose‐dependent manner (Fig. 1J,K). These findings indicate that leptin‐stimulated ATP generation in breast cancer cells was mediated by an increase in the utilization of fatty acid as a fuel source for ATP synthesis.

### 3.2 Autophagy is involved in induction of fatty acid oxidation and ATP production by leptin in breast cancer cells

**Fig. 2:**
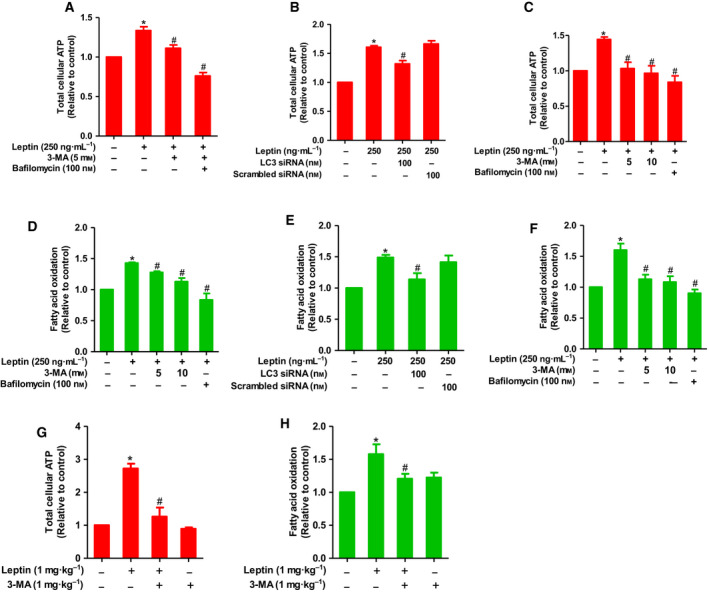
The role of autophagy in leptin‐induced ATP production and FAO in breast cancer cells. (A, B) MCF‐7 cells were pretreated with 3‐MA or Bafilomycin A1 at indicated concentrations for 1 h (A) or transfected with siRNA targeting LC3B for 48 h (B), followed by further stimulation with leptin for 24 h. ATP assay was then carried out as described in Materials and methods. (C) T47D cells were pretreated with 3‐MA or Bafilomycin A1 for 1 h followed by treatment with leptin for further 24 h, and cellular ATP levels were determined as described previously. (D, E) MCF‐7 cells were treated with leptin in the absence or presence of 3‐MA or Bafilomycin A1 (D) or transfected with LC3B siRNA (E). (F) T47D cells were treated with leptin in the absence or presence of 3‐MA or Bafilomycin A1. FAO level was determined by FAO assay as indicated in the methods. Values represent the fold change relative to the control cells and are expressed as mean ± SEM, *n* = 3. (G, H) MCF‐7 cell‐derived xenografts were developed in nude mice and treated with leptin (1 mg·kg^−1^) alone or in combination with 3‐MA (1 mg·kg^−1^) as mentioned in Materials and methods. (G) ATP levels were measured in tumor tissues and normalized to protein content in each sample. (H) MCF‐7 cells first separated from tumor tissues. Mitochondria were then isolated and used for the measurement of FAO according to the protocol described in Materials and methods. FAO levels were normalized to protein content in each sample. Values are presented as mean ± SEM, *n* = 5. *P < 0.05 compared to control group; ^#^ *P* < 0.05 compared to the group treated with leptin alone.

To elucidate the mechanisms underlying leptin‐stimulated ATP production, we examined if autophagy is implicated in ATP production by leptin. As shown in Fig. 2, pretreatment with 3‐MA and Bafilomycin A1, which are pharmacological inhibitors of autophagy, significantly suppressed leptin‐induced ATP production in both MCF‐7 (Fig. 2A) and T47D (Fig. 2C) breast cancer cells. The crucial role of autophagy induction in leptin‐induced ATP production was further confirmed by gene silencing of LC3B, in which transfection of siRNA targeting LC3B, but not scrambled control siRNA, abrogated the effect of leptin on cellular ATP level in MCF‐7 cells (Fig. 2B). Since FAO was required for ATP generation by leptin, we next examined the involvement of autophagy in leptin‐stimulated FAO activity. As shown in Fig. 2, suppression of autophagic flux using pharmacological inhibitors or transfection of siRNA targeting LC3B impeded FAO induction by leptin in both MCF‐7 (Fig. 2D,F) and T47D (Fig. 2E) breast cancer cells, which are essentially similar to the effect on ATP production.

To validate the *in vitro* effects of leptin on FAO‐dependent ATP production and the underlying role of autophagy activation *in vivo*, we generated MCF‐7 cell‐derived xenografts in BALB/c nude mice. After treatment of tumor bearing mice with leptin in the absence or presence of 3‐MA, we first examined the role of autophagy in leptin‐induced tumor growth in this model and found that leptin fueled breast tumor growth and this effect was significantly suppressed by cotreatment with the autophagy inhibitor 3‐MA, as determined by measurement of tumor growth, volume, and weight (Fig. S3A–D), confirming a critical contribution of autophagy induction to the tumor‐promoting effect of leptin in our experimental conditions. Effect of leptin on autophagy induction and the pharmacological effect of 3‐MA on autophagy induction were also verified (Fig. S3E). In subsequent experiments, as expected, leptin also elevated ATP production and FAO level in tumor tissues while cotreatment with 3‐MA abrogated induction of ATP biogenesis and FAO by this leptin (Fig. 2G,H). Taken together, these results imply that autophagy activation is required for FAO‐driven ATP production by leptin in breast cancer cells.

### 3.3 Autophagy activation contributes to fatty acid metabolic reprogramming by leptin in breast cancer cells

**Fig. 3:**
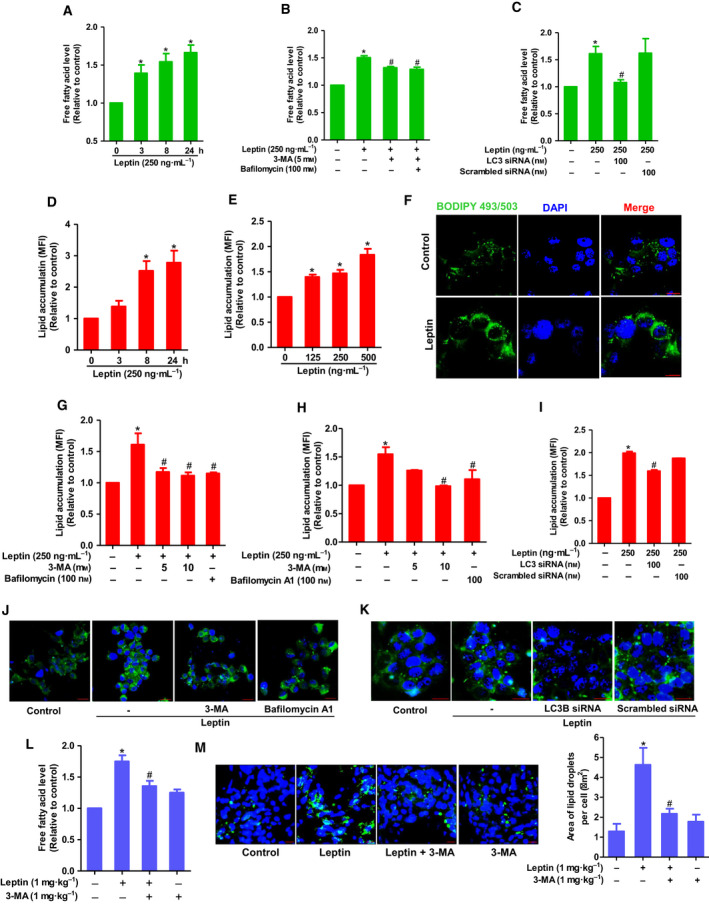
The role of autophagy in leptin‐induced fatty acid metabolic reprogramming in breast cancer cells. (A) MCF‐7 cells were treated with leptin (250 ng·mL^−1^) for the indicated time duration. (B, C) MCF‐7 cells were pretreated with 3‐MA or Bafilomycin A1 for 1 h (B) or transfected with siRNA targeting LC3B for 48 h (C), followed by stimulation with leptin for additional 24 h. Intracellular FFA level was determined as described in the Materials and methods. (D, E) MCF‐7 cells were treated with leptin (250 ng·mL^−1^) for the indicated time periods (D) or indicated concentrations for 24 h (E). Neutral lipid accumulation was measured by FACS analysis using fluorescent dye Bodipy 493/503 as described in Materials and methods. (F) MCF‐7 cells were treated with leptin (250 ng·mL^−1^) for 24 h followed by staining with Bodipy 493/503. The cells were then subjected to confocal microscopic analysis for detection of lipid droplets, which are indicated by green light. (G, H) MCF‐7 cells (G) or T47D cells (H) were pretreated with 3‐MA or Bafilomycin A1 for 1 h followed by further treatment with leptin for 24 h. Lipid accumulation was evaluated by FACS analysis. (I) MCF‐7 cells were transfected with LC3B siRNA for 48 h followed by treatment with leptin for additional 24 h. Lipid accumulation was evaluated by FACS analysis. (J, K) MCF‐7 cells were pretreated with 3‐MA or Bafilomycin A1 for 1 h (J) or transfected with LC3B siRNA for 24 h (K), followed by further stimulation with leptin for 24 h. Bodipy 493/503‐stained lipid droplets were visualized under confocal microscopy. For confocal analysis, representative images from three independent experiments have been shown. For FACS analysis, lipid accumulation levels were assessed though measuring mean fluorescence intensity (MFI) of Bodipy‐stained cells. Values represent fold change in comparison with the control group and are expressed as mean ± SEM, *n* = 3. (L, M) Tumor tissues were collected from nude mice bearing MCF‐7 xenograft tumor upon indicated treatments. (L) Equal amounts of tumor samples were homogenized and subjected to FFA assay as described above. FFA concentrations were normalized to protein content in each sample. (M) Tumor sections were prepared as described in Materials and methods section and stained with Bodipy 493/503. Lipid droplets were detected by confocal microscopic analysis. Representative images for each group are presented in left panel. Total area of lipid droplets normalized to cell number was determined using imagej software [71] and shown in right panel. Green light indicates lipid droplet and blue light indicate DAPI staining, scale bar: 20 µm. Values are presented as mean ± SEM, *n* = 5. *P < 0.05 compared to control group; ^#^ *P* < 0.05 compared with the group treated with leptin only.

During FAO, FFA released by lipid catabolism is broken down into acyl‐CoA molecules for energy production. We tested whether leptin could increase the abundance of intracellular FFA to provide breast cancer cells with substrates for β‐oxidation. As shown in Fig. 3A, treatment with leptin enhanced the intracellular FFA level. Furthermore, leptin‐induced FFA release was significantly suppressed by 3‐MA or Bafilomycin A1 pretreatment (Fig. 3B), or LC3B gene silencing (Fig. 3C), suggesting that autophagy induction is implicated in intracellular FFA generation by leptin in breast cancer cells.

Given that intracellular lipid contents are mainly stored as neutral lipids called lipid droplets that may serve as a reservoir of FFA, we next investigated the effects of leptin on neutral lipid accumulation using flow cytometry analysis. Leptin increased lipid accumulation in a time‐ and dose‐dependent manner in MCF‐7 breast cancer cells (Fig. 3D,E). Induction of lipid droplet formation by leptin was further confirmed by confocal microscopic imaging (Fig. 3F). In addition, leptin‐induced lipid accumulation was significantly mitigated by pretreatment with 3‐MA or Bafilomycin A1, as determined by flow cytometry (Fig. 3G,H) and confocal microscopic analyses (Fig. 3J). Likewise, inhibition of autophagy by LC3B gene silencing also restored the promoting effect of leptin on intracellular lipid reservation (Fig. 3I,K). Finally, we verified the contributions of autophagy to leptin‐induced alterations in lipid metabolism in a mouse model of xenograft tumor. In consistent with the *in vitro* data, leptin increased FFA level and induced lipid accumulation in tumor tissues, both of which were almost completely suppressed by cotreatment with 3‐MA (Fig. 3L,M), confirming the crucial role of autophagy induction in lipid metabolic reprogramming by leptin.

### 3.4 Leptin upregulates SREBP‐1 via activation of PI3K signaling and autophagy in breast cancer cells

**Fig. 4:**
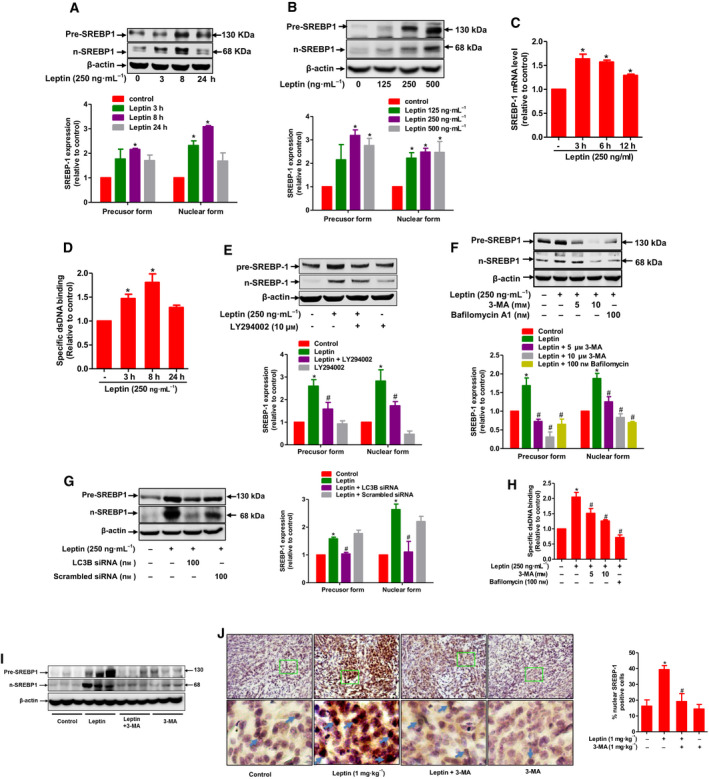
The role of autophagy in SREBP‐1 induction in breast cancer cells. (A, B) MCF‐7 cells were treated with leptin (250 ng·mL^−1^) for indicated time periods (A) or different concentrations of leptin for 5 h (B). Total protein lysates were prepared and used for the measurement of precursor and nuclear SREBP‐1 expression by western blot analysis. (C) MCF‐7 cells were treated with leptin (250 ng·mL^−1^) for indicated time periods and mRNA level of SREBP‐1 was determined by RT‐qPCR. (D) MCF‐7 cells were treated with leptin for indicated time duration. Nuclear extracts were prepared and used for determining the specific DNA binding activity of SREBP‐1. (E–G) MCF‐7 cells were pretreated with LY294002 (E) or 3‐MA and Bafilomycin A1 (F) for 1 h or transfected with LC3B siRNA for 48 h (G), followed by further incubation with leptin for 5 h. Precursor and nuclear SREBP‐1 expression levels were determined by western blot analysis. Representative images from at least three independent experiments are presented. β‐actin was served as a loading control. Relative band intensities of the target proteins compared to the loading control were quantified by densitometric analysis and are presented as lower bar diagrams shown in the lower panel of each figure. (H) MCF‐7 cells were pretreated with 3‐MA or Bafilomycin A1 for 1 h, followed by further incubation with leptin for 5 h. Nuclear extracts were prepared and used for determining the specific DNA binding activity of SREBP‐1. Values are expressed as the fold change compared with control and are presented as mean ± SEM, *n* = 3 (I) Total cellular extracts from tumor tissues from mice treated with leptin and/or 3‐MA as well as control mice were examined for the expression of SREBP‐1. (J) Tumor sections were prepared and subjected to immunochemistry staining for detection of SREBP‐1. Representative images for each group were presented in left panel and the percentage of staining positive cells was shown in right panel; scale bar: 5 µm. Values are presented as mean ± SEM, *n* = 5. *P < 0.05 compared to control; ^#^ *P* < 0.05 compared with the group treated with leptin only.

Cancer cells prefer *de novo* fatty acid synthesis despite abundance of exogenous lipid in the tumor microenvironment [36]. To investigate whether leptin induces lipogenesis in breast cancer cells, we examined the effect of leptin on the expression of SREBP‐1, a master transcriptional regulator of fatty acid biosynthesis [37]. We observed that leptin markedly increased expression of both precursor and cleaved forms of SREBP‐1 in a time‐ and dose‐dependent manner in MCF‐7 cells (Fig. 4A,B). Leptin also induced increase in SREBP‐1 mRNA expression (Fig. 4C). Moreover, we also found that leptin substantially enhanced the amount of nuclear SREBP‐1 associated with specific dsDNA sequences containing the SREBP‐1 response element (SRE) (Fig. 4D), indicating an increase of SREBP‐1 in SRE binding activity upon leptin stimulation. Since leptin has been known to activate PI3K/Akt signaling pathway which is closely connected to SREBP‐1 induction [38], we sought to examine contribution of this signaling pathway to leptin‐induced SREBP‐1 expression. We first confirmed that leptin enhances phosphorylation of Akt in a PI3K‐dependent manner in MCF‐7 cells (Fig. S1). Importantly, blockade of PI3K signaling with LY294002, a pharmacological inhibitor of PI3K, led to partial abrogation of leptin‐stimulated SREBP‐1 expression in MCF‐7 cells (Fig. 4E), suggesting that PI3K/Akt signaling is required for SREBP‐1 induction by leptin. Furthermore, given the fact that autophagy was critical for elevated intracellular lipid pool by leptin, we next examined the role of autophagy in SREBP‐1 induction. As shown in Fig. 4F,G, inhibition of autophagy induction by treatment with pharmacological inhibitors (3‐MA and Bafilomycin A1), or transfection with siRNA targeting LC3B, abrogated the promoting effect of leptin on SREBP‐1 expression. In addition, leptin‐induced increase in the specific DNA binding activity of SREBP‐1 was also significantly inhibited by treatment with autophagy inhibitors (Fig. 4H). It is worth noting that while both PI3K signaling and autophagy are involved in SREBP‐1 induction by leptin, autophagy induction is found to be independent of PI3K signaling. Cotreatment leptin with PI3K inhibitor did not result in restoration of autophagy‐related genes but further increases their expression levels in MCF‐7 cells (Fig. S2). These findings demonstrate that autophagy induction and PI3K signaling are separate pathways and modulate SREBP‐1 induction through distinct signaling mechanisms. We finally confirmed the role of autophagy induction in leptin‐promoted SREBP‐1 expression in the *in vivo* condition. In line with the *in vitro* findings, leptin administration upregulated precursor SREBP‐1 and induced SREBP‐1 maturation in tumor tissues, while cotreatment with 3‐MA prominently attenuated these changes (Fig. 4I). Likewise, immunohistochemistry images depicted a substantial increase in expression of nuclear SREBP‐1 upon treatment with leptin, which was also mitigated by co‐administration with 3‐MA (Fig. 4J). Together, these results reveal that leptin drives the expression, maturation, and SRE binding activity of SREBP‐1 through activation of autophagy and PI3K.

### 3.5 SREBP‐1 mediates the effects of leptin on fatty acid metabolism in breast cancer cells

**Fig. 5:**
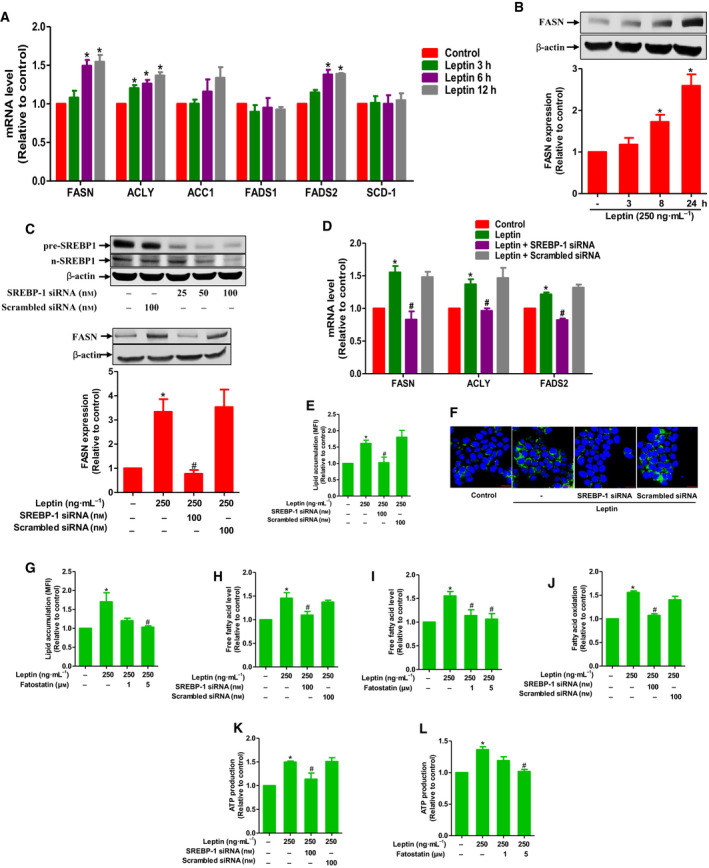
The role of SREBP‐1 in alterations of the fatty acid metabolism induced by leptin in breast cancer cells. (A, B) MCF‐7 cells were treated with leptin for indicated time periods. Messenger RNA levels of genes of interest were analyzed using RT‐qPCR (A). FASN protein expression was measured by western blot analysis (B). (C) MCF‐7 cells were transfected with siRNA targeting SREBP‐1 followed by further stimulation with leptin for 24 h. (Upper panel) Gene silencing efficiency was monitored by western blot analysis after 48 h of transfection. (Lower panel) Protein expression level of FASN was measured by western blot analysis. (D) MCF‐7 cells were transfected with siRNA targeting SREBP‐1 followed by further stimulation with leptin for 24 h. Messenger RNA levels of the potential target genes were determined by RT‐qPCR. (E–L) MCF‐7 cells were transfected with siRNA targeting SREBP‐1 for 48 h (E, F, H, J, and K) or pretreated with fatostatin (G, I, and L) for 1 h before being incubated with leptin for additional 24 h. (E–G) Lipid accumulation was evaluated by FACS analysis (E, G) and confocal microscopy (scale bar: 20 µm) (F). Representative images from three independent experiments have been presented. (H, I) Intracellular FFA level was determined as described in Materials and methods. (J) FAO assay was performed to measure fatty acid consumption rate. (K, L) Cellular ATP level was analyzed as described in Materials and methods. Values are expressed as the fold change compared with the control and are presented as mean ± SEM, *n* = 3. *P < 0.05 compared to control; ^#^ *P* < 0.05 compared with the cells treated with leptin only.

SREBP‐1 activates a wide range of genes participating in fatty acid synthesis. Since leptin increased the expression and activity of SREBP‐1, we questioned if leptin modulates the genes related with fatty acid synthesis in breast cancer cells. Of the various genes implicated in fatty acid synthesis, leptin significantly enhanced mRNA levels of fatty acid synthase (*FASN*), ATP citrate lyase (*ACLY*), and fatty acid desaturase 2 (*FADS2*) (Fig. 5A), while no significant effect on *ACC1*, *FADS1*, and *SCD‐1* expression was observed. Leptin‐induced FASN expression was further confirmed by western blot analysis (Fig. 5B). In subsequent experiments to verify the functional role of SREBP‐1, transient knockdown of SREBP‐1 led to restoration of FASN protein expression (Fig. 5C), as well as *FASN*, *ACLY*, and *FADS2* mRNA expression (Fig. 5D) in MCF7 cells treated with leptin. Intriguingly, blockade of SREBP‐1 signaling by siRNA transfection also mitigated leptin‐simulated lipid accumulation as determined by flow cytometry (Fig. 5E) and confocal microscopic analyses (Fig. 5F). Similar results were observed following treatment with fatostatin (Fig. 5G), a pharmacological inhibitor of SREBPs, suggesting that SREBP‐1 critically contributes to induction of fatty acid synthesis under leptin stimulation in breast cancer cells. Although lipogenesis and lipolysis are considered as opposite processes in which decreased lipid accumulation is often accompanied by increased lipolysis, it is interesting to note that transient knockdown of SREBP‐1 and treatment with fatostatin also suppressed fatty acid release (Fig. 5H,I) and fatty acid consumption as measured by FAO assay (Fig. 5J) in cells treated with leptin. Furthermore, leptin‐induced ATP production was also impeded by SREBP‐1 siRNA transfection or treatment with fatostatin (Fig. 5K,L). Taken together, these findings reveal an integral role of SREBP‐1 in alterations in lipid metabolism induced by leptin.

### 3.6 SREBP‐1 mediates the effect of leptin on breast cancer cell growth

**Fig. 6:**
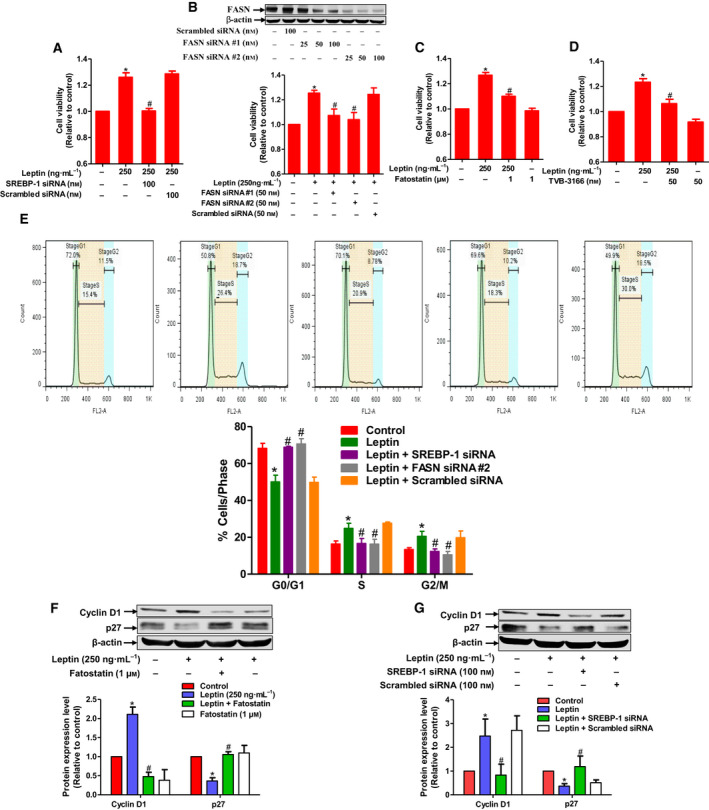
The role of SREBP‐1 induction in leptin‐induced growth of breast cancer cells. (A–D) (A) MCF‐7 cells were transfected with siRNA targeting SREBP‐1 for 48 h followed by further incubation with leptin for additional 48 h. (B) Cells were transfected with siRNA targeting FASN for 48 h followed by further incubation with leptin for additional 48 h. (Upper panel) gene silencing efficiency was monitored by western blot analysis after 48 h of transfection. (C, D) Cells were pretreated with fatostatin (C) or TVB‐3166 (D) for 1 h followed by further incubation with leptin for additional 48 h. Cell viability was assessed by MTS assay as described in Materials and methods. (E) MCF‐7 cells were transfected with siRNA targeting SREBP‐1 or FASN for 48 h followed by further incubation with leptin for additional 48 h. Cells were then subjected to cell cycle analysis. The representative image for populations of the cells in different phases of the cell cycle was shown in upper panel and the average percentage of the cells distributed in each phase of cell cycle from three independent experiments was presented in lower panel. (F, G) MCF‐7 cells were pretreated with fatostatin (F) or transfected with SREBP‐1 siRNA (G) followed by further stimulation with leptin for additional 48 h. Expression levels of cyclin D1 and p27 were determined by western blot analysis. Representative images from at least three independent experiments were presented. Relative band intensities of the target proteins were measured by densitometric analysis and presented in the lower panel of each figure. β‐actin was used as loading control. Values are expressed as the fold change compared with control and are presented as mean ± SEM, *n* = 3. *P < 0.05 compared to control; ^#^ *P* < 0.05 compared with the cells treated with leptin only.

Accumulating evidence suggests that fatty acid metabolic reprogramming modulates survival and growth of various cancer cells. We next explored a potential link between changes in fatty acid metabolism and well‐characterized effects of leptin on breast tumor growth. Gene silencing of SREBP‐1 or FASN abrogated the leptin‐enhanced MCF‐7 cell viability (Fig. 6A,B). Consistently, pretreatment with fatostatin or TVB‐3166, which are pharmacological inhibitors of SREBP‐1 and FASN, respectively, also impeded cancer cell growth promoted by leptin (Fig. 6C,D). In addition, enhanced cell populations in S and G2/M phase, but decreased G0/G1, induced by leptin treatment were significantly affected by gene silencing of SREBP‐1 or FASN (Fig. 6E), indicating a crucial role of SREBP‐1 in cell cycle progression stimulated by leptin. It has been reported that leptin promotes G1/S phase cell cycle transition through upregulation of cyclin D1, a positive regulator of the cell cycle, but suppression of p27^Kip1^, a potent inducer of cell cycle arrest [39]. Herein, we showed that blockade of SREBP‐1 signaling led to mitigation of cyclin D1 induction and restoration of p27^Kip^ expression in leptin‐treated cells (Fig. 6F,G). Collectively, these data suggest that alterations in leptin‐mediated fatty acid metabolism may contribute to the promoting effect of leptin on breast cancer cell growth.

**Fig. 7:**
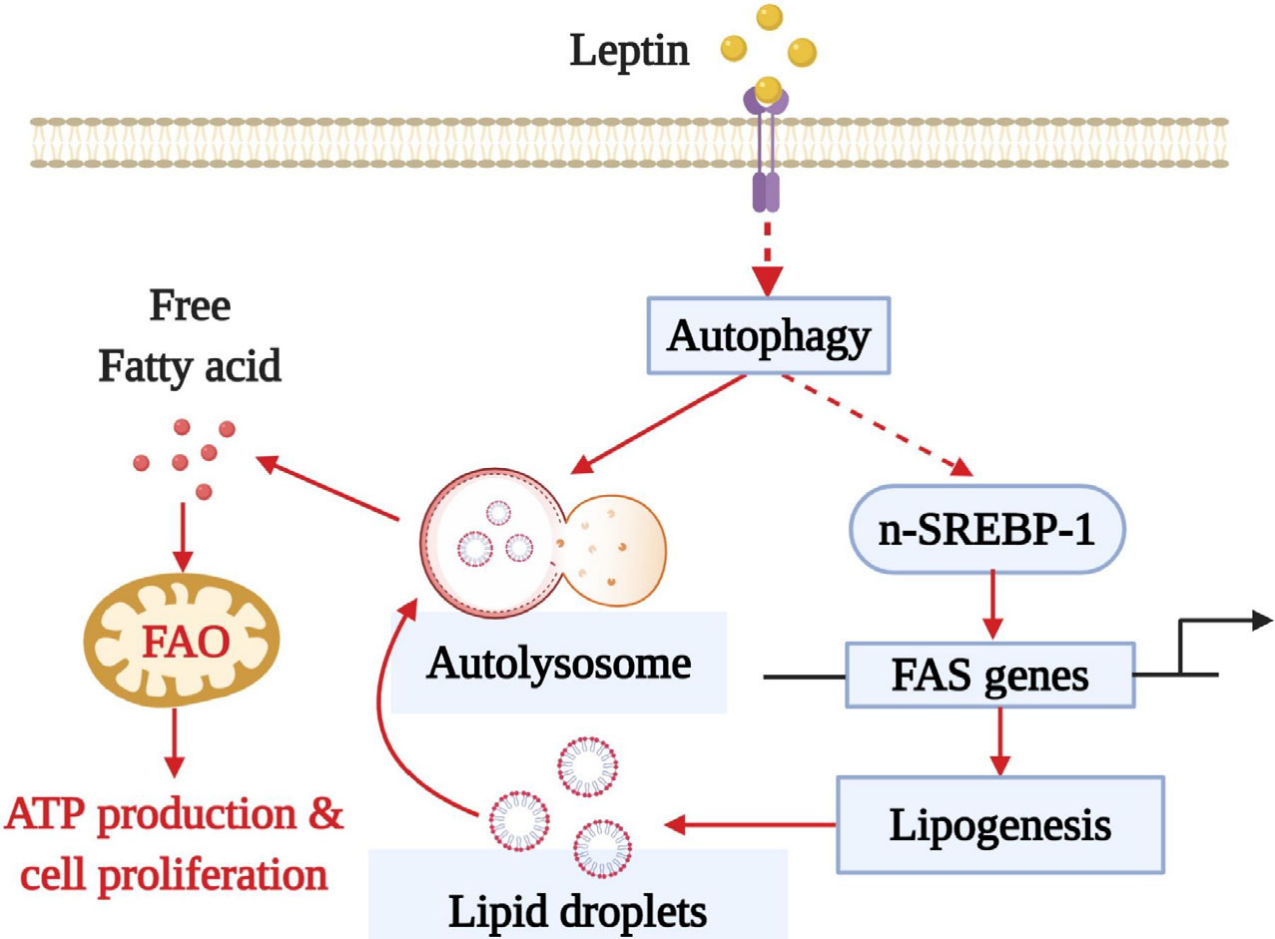
Proposed model for the role of autophagy activation in fatty acid metabolic reprogramming in breast cancer cells and the promotion of tumor growth by leptin. Leptin has been shown to induce growth of breast cancer cells via autophagy induction. In this study, we further elucidate the mechanisms by which autophagy contributes to leptin‐induced growth of breast cancer cell and found that autophagy activation plays a crucial role in the changes of fatty acid metabolism, which is required for breast tumor growth. Autophagy induction contributes to an extensive reprogramming of cellular fatty acid metabolism in breast cancer cells stimulated with leptin, as evidenced by a concomitant increase in fatty acid synthesis (FAS) and FAO. On the one hand, leptin‐stimulated autophagy leads to activation of SREBP1, a master regulator of fatty acid synthesis, which in turn enhances synthesis and accumulation of fatty acid in intracellular structures called lipid droplets through transcriptional induction of FAS genes. However, the molecular mechanisms by which autophagy causes SREBP‐1 induction remain elusive. On the other hand, autophagy activation promotes the use of fatty acid for energy production via stimulation of FFA liberation. The elevation of FAO level critically contributes to increased ATP production by leptin that facilitates breast tumor growth by satisfying the high need of energy in cancer cells. Given that fatty acid metabolism critically contributes to growth of cancer cells, targeting autophagy and SREBP‐1 induction would be a potential therapeutic intervention for breaking the linkage between adiposity‐leptin and breast cancer.

## 4 Discussion

A large body of recent evidence clearly indicates that adipose tissue acts as a potential mediator in the development and progression of various types of cancers [40]. The modulatory role of adipose tissue in tumors is mediated by adipose tissue‐derived cytokines, collectively called adipokines, which may act in a paracrine or endocrine manner. Of the diverse adipokines, adiponectin and leptin represent the pleiotropic adipokine class predominantly produced by adipocytes. Interestingly, adiponectin and leptin exhibit opposite roles in tumor growth. While adiponectin possesses potent antitumor activities via various mechanisms [35, 41], leptin has been found to be correlated with cancer aggressiveness and promote tumor growth, either directly via oncogenic signaling or indirectly through regulation of immune response and angiogenesis [41, 42, 43]. A recent study has also reported that cellular metabolic reprogramming contributes to leptin‐induced proliferation and survival of cancer cells [31]. However, little is known about the effects of leptin on detailed metabolic reprogramming and the underlying molecular mechanisms mostly remain elusive. In the present study, we have shown that leptin increases ATP generation by inducing a profound reprogramming in fatty acid metabolism, including FAO and FAS, in estrogen receptor ER‐positive breast cancer cells. In addition, we have demonstrated for the first time that SREBP‐1 induction, mediated via autophagy, plays a critical role in leptin‐induced metabolic alterations in cancer cells.

Tumor tissues are composed of rapidly proliferating malignant cells with a higher demand for energy and cellular building blocks, such as nucleic acids, proteins, and lipids. The high metabolic rate of cancer cells results in dramatic changes in the tumor microenvironment, which in turn require cellular metabolic rewiring to adapt to unfavorable conditions for cell growth, including acidosis, nutrient deprivation, and hypoxia [44, 45, 46]. Surprisingly, tumor cells have been long considered to mainly rely on glycolysis instead of mitochondrial respiration for energetic production from glucose [11]. However, because of low efficiency of the glycolytic pathway, this pathway alone may not sufficiently satisfy the greater need for energy in cancer cells [47]. Recently, emerging evidence has highlighted a critical role of fatty acid β‐oxidation in survival, proliferation, and chemoresistance of breast cancer cells, suggesting that fatty acid can be used by cancer cells as an alternative fuel for ATP biosynthesis [16, 48]. Herein, we found that leptin enhanced cellular ATP levels in ER‐positive breast cancer cells that showed a high dependence on oxidative phosphorylation but not glycolytic pathway. In addition, inhibition of FAO led to restoration of ATP levels in leptin‐treated breast cancer cells (Fig. 1G), indicating that leptin stimulates ATP generation in FAO‐dependent manner. Interestingly, neither ATP levels (Fig. 1D) nor cell growth (Fig. S4) was affected by leptin in MDA‐MB 231 estrogen receptor (ER) negative breast cancer cells, supporting the hypothesis that ATP production and FAO‐mediated via ER‐dependent mechanisms is required for leptin‐induced growth of breast cancer cell. Previous studies indicated that leptin increased oxygen consumption rate and promoted mitochondrial β‐oxidation in cancer cells [30]. However, it remains controversial whether FAO induction by this adipokine leads to an elevation of total cellular ATP levels or it merely reflects a shift from ATP production via glycolysis to that based on mitochondrial oxidative phosphorylation [30, 31, 32]. In the present study, elevated FAO and ATP generation appears to occur concomitantly, strongly suggesting a close interrelation between these two outcomes in leptin‐stimulated breast cancer cells. However, given that leptin stimulation may affect many other possible metabolic processes, including improved mitochondrial function and decreased glucose‐dependent energetic production [31], other metabolic pathways could be included in the growth of cancer cells by leptin. Further studies will be needed to gain better insights into the role of cancer cell‐specific metabolism in tumor growth by leptin.

Autophagy has long been recognized as a critical regulator of cellular metabolism [49]. Interestingly, while previous studies have demonstrated that leptin induces growth of breast cancer cells in an autophagy‐dependent manner [6], whether autophagy contributes to leptin‐induced metabolic alterations has never been assessed. Considering the coexistence of autophagy activation and elevated FAO, we speculated that induction of FAO and ATP production by leptin in breast cancer cells might be ascribed to activation of autophagy. As expected, we observed that inhibition of autophagy by pharmacological inhibitors or knockdown of autophagy‐related LC3B genes resulted in restoration of ATP levels and FAO rates in leptin‐treated breast cancer cells (Fig. 2), indicating the crucial role of autophagy in FAO induction and ATP production. In addition, since leptin‐enhanced intracellular FFA levels were counteracted by autophagy suppression (Fig. 3), we presume that this adipokine induces lipolysis via activation of autophagy to increase the abundance of intracellular FFAs facilitating FAO‐mediated energetic generation. With regard to the mechanisms underlying FAO induction by leptin, Wang *et al*. [50] recently reported that CPT1B upregulation is involved in leptin‐stimulated FAO induction in breast cancer stem cells. However, little is known about the molecular mechanisms involved. Herein, we demonstrated for the first time that autophagy induction critically contributes to FAO induction by leptin and further elucidated the mechanisms by which leptin mobilizes FFAs from intracellular lipid depots to use for FAO.

Cellular lipid homeostasis is controlled by balancing between lipid formation (lipogenesis) and catabolism (lipolysis). While the lipolytic process contributes to a major source of oxidative substrates for tumor cell respiration, lipogenesis provides cells with building blocks for membrane biogenesis, post‐translational modification of proteins, and lipid‐based signaling transduction, and is required for maintenance of intracellular lipid depots. Therefore, lipogenesis is also supposed to be essential for cancer cell survival [51, 52]. In most normal cells, increased FAO is often coupled with decreased lipogenesis and lipid accumulation. However, in some cases, both lipolysis and lipogenesis can occur simultaneously in cancer cells under certain conditions. For instance, chronic acidosis in the tumor microenvironment not only promotes metabolic rewiring in cancer cells toward FAO, but also supports fatty acid biosynthesis from glutamine [15]. In addition, fatty acid synthesis and degradation were reported to co‐exist in breast cancer cells that plays an important role in regulation of cellular oxidative stress [53]. Likewise, although autophagy has long been considered a common catabolic pathway at the subcellular level, recent evidence has indicated a positive correlation between autophagy activation and lipogenesis [27]. Driven by these considerations, we examined if autophagy activation is implicated in alterations in lipogenesis and intracellular lipid deposits by leptin and elucidated the underlying mechanisms. Herein, we found that leptin enhanced formation of lipid droplets in breast cancer cells and autophagy inhibition mitigated this lipid accumulation (Fig. 3), indicating that autophagy might be responsible for leptin‐induced lipogenesis.

In this study, leptin was found to induce lipolysis and intracellular lipid accumulation, both of which were mediated by autophagy activation. While autophagy has been widely known to directly contribute to lipid degradation, the mechanisms by which this process modulates lipogenesis remain poorly characterized. In an attempt to clarify how leptin‐stimulated autophagy induces lipid accumulation in breast cancer cells, we showed that leptin upregulated and activated SREBP‐1 in ER‐positive breast cancer cells as well as in tumor tissues. It is surprising that while leptin has been well known as a suppressor of SREBP‐1 in liver and adipose tissues [54, 55], little is known about contributions of SREBP‐1 to the effects of leptin on cancer‐specific metabolism and tumor growth. Interestingly, a recent study has been suggested that leptin activates SREBP‐2 and thereby promotes migration and invasion of breast cancer cells via upregulation of acetyl‐CoA acetyltransferase 2 (ACAT2) [38]. Hence, it could be reasoned that modulatory effects of leptin on SREBP is determined by context‐dependent manners and SREBPs induction by leptin would be specific for cancer cells, which may play a key role in its tumor‐promoting effects. Mechanistically, we found that both autophagy and PI3K/Akt signaling are involved in activation of SREBP‐1 by leptin. In fact, it has been previously reported that leptin modulates expression of SREBPs though PI3K/Akt pathways [38], whereas autophagy induction might be a new mechanism underlying SREBP‐1 upregulation by this adipokine. Previous studies also highlighted the linkage between autophagy and SREBPs. For example, a Gene Ontology (GO) analysis showed that both SREBP‐2 and autophagy‐related genes were upregulated in response to starvation [56]. Likewise, autophagy activation by rapamycin led to enhanced expression of SREBP‐1 in pancreatic cancer cells [28]. These findings suggest an interesting connection between autophagy and different isoforms of SREBPs.

Given the role of SREBP‐1 as a master regulator for the fatty acid synthesis pathway [57], we speculated that induction of SREBP‐1 may critically contribute to leptin‐stimulated lipid metabolic reprogramming. As expected, blockade of SREBP‐1 signaling by fatostatin, which inhibits SREBPs activation by disrupting the movement of SREBPs to the Golgi, resulted in abrogation of both leptin‐induced intracellular lipid accumulation and fatty acid consumption (Fig. 5). While fatostatin is the most commonly used pharmacological inhibitor of SREBP, nonspecific effect of this compound has been reported. To minimize the nonspecific effects, we tried to keep concentration of fatostatin as low as possible (1 μm). Furthermore, taking into account potential off‐target effects of fatostatin as previously reported [58], the role of SREBP‐1 was further confirmed by gene silencing of SREBP‐1 (Fig. 5), allowing to conclude that SREBP‐1‐mediated lipogenesis is required for FAO stimulation by leptin in breast cancer cells. For the fatty acid metabolism, leptin has been shown to upregulate CD36, a scavenger receptor functioning as a transporter of long‐chain fatty acids, in various normal and cancer cells, which in turn facilitates uptake of exogenous fatty acids [59]. Data demonstrated in the present study reveal a novel mechanism by which leptin increases the supply of fatty acid for energetic generation and other metabolic needs of breast cancer cells. To date, at least 30 genes have been identified whose expressions are regulated by SREBPs family. However, dependence of individual genes on SREBP‐1 might be different among cancer cell types and dependent on the inducers and inhibitors used [20, 60, 61]. Herein, taking into account that leptin induces SREBP‐1 in MCF‐7 breast cancer, we have identified SREBP‐1 target genes in response to leptin stimulation, including FASN, ACLY, and FADS2 (Fig. 5). Although all of these genes have been linked to metabolic functions, most attention related to the growth and invasion of cancer cells has been directed to FASN, which is often overexpressed in tumor tissues and represents the lipogenic phenotype in cancer pathogenesis [62, 63, 64, 65]. Earlier studies demonstrated that *de novo* lipogenesis was essentially required for the growth of various tumors, such as melanoma, pancreatic, and colon cancers [13, 20, 66]. Overexpression of SREBP‐1 was also observed in breast tumor tissues, which may be considered as an independent prognostic marker in breast cancer [67]. On the contrary, SREBP‐1 was reported to promote p21 expression whereby it induced cell growth arrest in Saos‐2 cells, a human osteosarcoma cell line [68]. These contrasting observations could be due to different dependence of cancer cells on endogenous fatty acid synthesis as previously suggested [69]. Given the crucial role of SREBP‐1 induction and subsequent fatty acid metabolism in cancer cell growth, we performed a series of *in vitro* experiments to elucidate the potential contribution of SREBP‐1 and FASN to leptin‐stimulated growth of breast cancer cells, and clearly demonstrated that SREBP‐1 activation and FASN induction mediates the promoting actions of leptin on cancer cell growth (Fig. 6). As a downstream target of autophagy induction, we assume that SREBP‐1 and FASN may play a role as a linker between leptin‐induced autophagy and tumorigenesis [6]. Accumulating evidence supports that targeting *de novo* fatty acid synthesis is likely to become a new therapeutic approach for the treatment of cancer [51]. With regard to the role of leptin in the crosstalk between obesity and breast cancer as aforementioned, our findings suggest that suppression of SREBP‐1‐dependent lipogenesis is a potential intervention for disrupting obesity‐cancer axis. It is important to note that while this study mainly focuses on the relationship between leptin modulation of fatty acid metabolism and cellular energy production, both fatty acid synthesis and FAO contribute to other essential cellular functions, which should be taken into consideration for justifying the impact of leptin‐driven fatty acid metabolic rewiring on tumor growth. In fact, FAO generates a large number of acetyl‐CoA molecules for ATP production in Krebs cycle; however, acetyl‐CoAs also play a crucial role in post‐translational protein acetylation and lipogenesis [70]. Therefore, a modest increase in cellular ATP levels as observed in this study may result from the coupling between FAO and lipogenesis, as well as other uses of acetyl‐CoAs other than energy production. Further studies will be needed to provide a better insight into how leptin‐induced alterations in fatty acid metabolism fuel the growth of breast cancer cells.

## 5 Conclusions

In summary, the present study clearly demonstrates that leptin induces fatty acid metabolic changes in breast cancer cells, which are characterized by a concomitant increase in FAO and FAS, and further provides the first evidence that autophagy activation drives this fatty acid metabolic reprogramming (Fig. 7). Data presented in this study suggest a mechanistic model for coexistence of two of these opposite processes by leptin stimulation. First, autophagy induction promotes lipolysis and increases FFA release, which in turn stimulates β‐oxidation of fatty acid and FAO‐dependent ATP production. Second, autophagy activation induces the expression and maturation of SREBP‐1 that might be responsible for upregulation of *de novo* biosynthesis of fatty acid and enhanced intracellular lipid accumulation. Finally, we showed for the first time that induction of SREBP‐1 and its target gene, FASN, plays a key role in the tumor‐promoting effects of leptin. However, it remains an open question as to which metabolic pathway provides substrates for leptin‐stimulated lipogenesis. In addition, future studies are needed to further elucidate the mechanism by which lipid metabolic rewiring modulates breast tumor growth by leptin.

## Conflict of interest

The authors declare no conflict of interest.

## Author contributions

P‐HP and NTP designed the study, performed the experiments. P‐HP, D‐VP, and NTP analyzed the data. P‐HP and D‐VP wrote the manuscript. P‐HP contributed funding to purchase materials and reagents.

### Peer Review

The peer review history for this article is available at https://publons.com/publon/10.1002/1878‐0261.12860.

## Supporting information


**Fig. S1.** The role of PI3K in leptin‐stimulated Akt phosphorylation in MCF‐7 cells.

**Fig. S2.** The effect of LY294002 on autophagy induction by leptin in MCF‐7 cells.

**Fig. S3.** The crucial role of autophagy activation in the breast cancer cell growth induced by leptin in a tumor xenograft model.

**Fig. S4.** The effect of leptin on cell viability of MDA‐MB‐231 breast cancer cells.

**Table S1.** Sequences of siRNA duplexes and PCR primers used in the study.
