# Supplementary material for: Autophagy activation and SREBP‐1 induction contribute to fatty acid metabolic reprogramming by leptin in breast cancer cells
Source: Mol Oncol. 2020 Dec 5;15(2):657–78. doi: 10.1002/1878-0261.12860 (PMC7858107; doi:10.1002/1878-0261.12860)
Supplement: Supplementary file 1 — Fig. S1. The role of PI3K in leptin‐stimulated Akt phosphorylation in MCF‐7 cells. [file MOL2-15-657-s001.pdf]

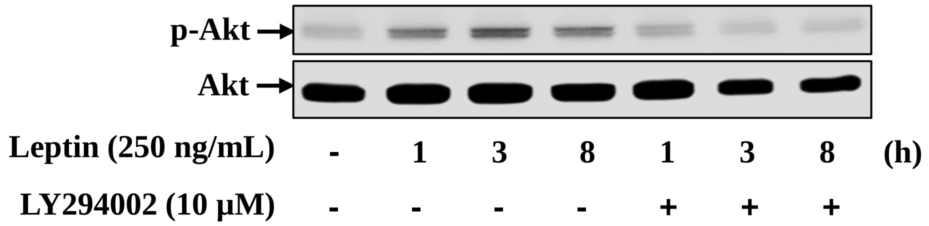

Fig. S1. *The role of PI3K in leptin-stimulated Akt phosphorylation in MCF-7 cells*. MCF-7 cells were pretreated with LY294002 (10 μM) for 1 h, followed by incubation with leptin (250 ng/ml) for further 1, 3 or 8 h. Total and phosphorylated Akt levels were determined by western blot analysis.
