# Supplementary material for: Autophagy activation and SREBP‐1 induction contribute to fatty acid metabolic reprogramming by leptin in breast cancer cells
Source: Mol Oncol. 2020 Dec 5;15(2):657–78. doi: 10.1002/1878-0261.12860 (PMC7858107; doi:10.1002/1878-0261.12860)
Supplement: Supplementary file 2 — Fig. S2. The effect of LY294002 on autophagy induction by leptin in MCF‐7 cells. [file MOL2-15-657-s002.pdf]

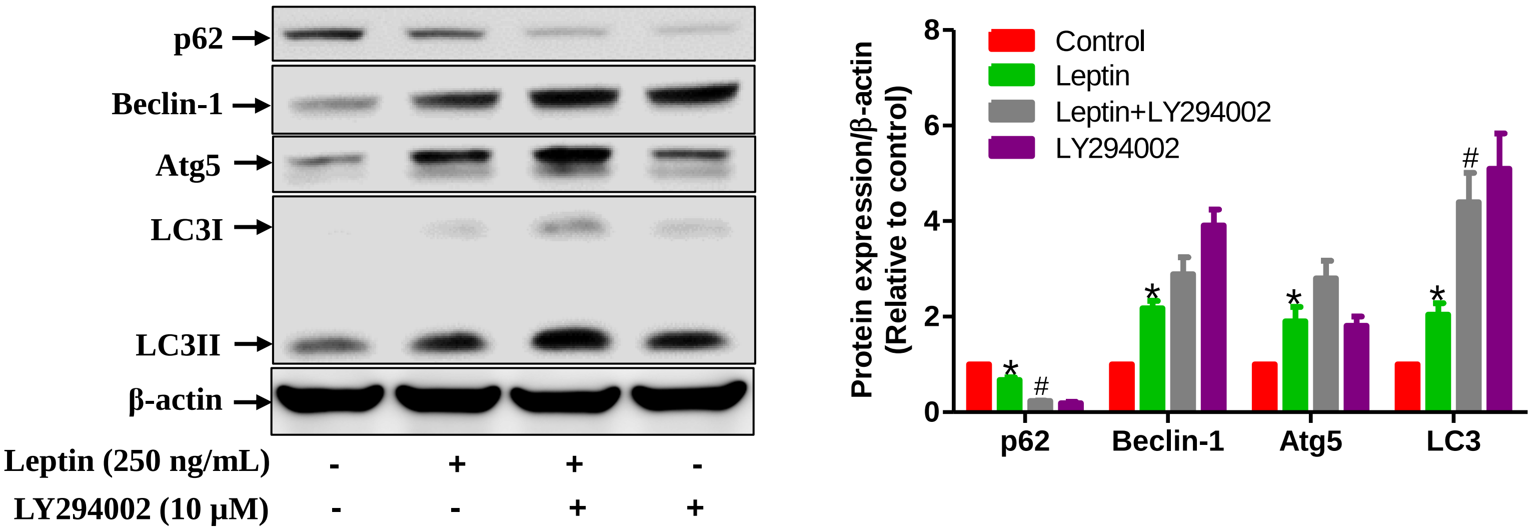

Fig. S2. *The effect of LY294002 on autophagy induction by leptin in MCF-7 cells*. MCF-7 cells were pretreated with LY294002 (10 μM) for 1 h, followed by incubation with leptin (250 ng/ml) for further 8 h. The expression levels of p62, Beclin-1, Atg5 and LC3 were examined by Western blot analysis. Representative images from at least three independent experiments are presented. β-actin was served as a loading control. Relative band intensities of the target proteins compared to the loading control were quantified by densitometric analysis and are presented as bar diagrams shown in the right panel.
