# Supplementary material for: Autophagy activation and SREBP‐1 induction contribute to fatty acid metabolic reprogramming by leptin in breast cancer cells
Source: Mol Oncol. 2020 Dec 5;15(2):657–78. doi: 10.1002/1878-0261.12860 (PMC7858107; doi:10.1002/1878-0261.12860)
Supplement: Supplementary file 3 — Fig. S3. The crucial role of autophagy activation in the breast cancer cell growth induced by leptin in a tumor xenograft model. [file MOL2-15-657-s003.pdf]

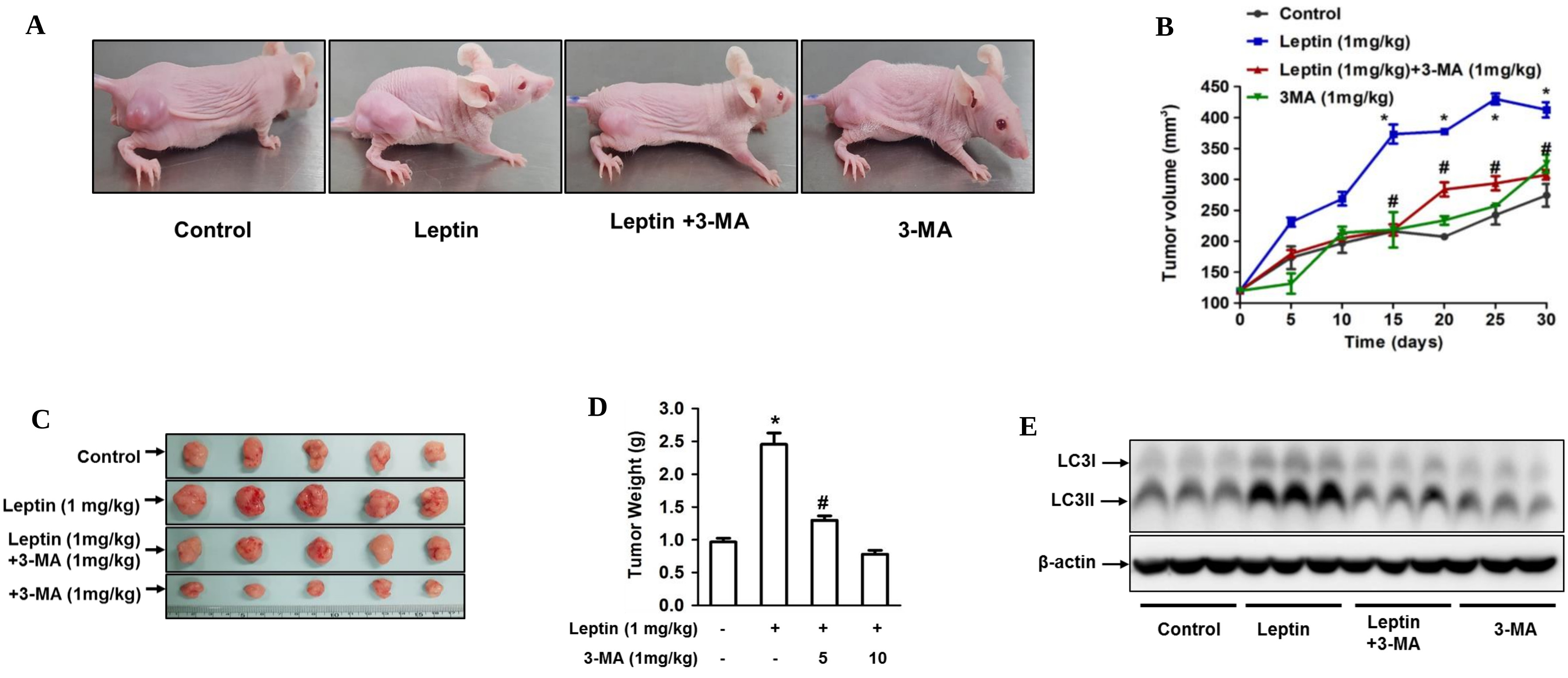

Fig. S3. *The crucial role of autophagy activation in the breast cancer cell growth induced by leptin in a tumor xenograft model.* MCF-7 xenografted tumors were generated in 4–week old BALB/c nude male mice by subcutaneous injection of 107 MCF-7 cells into the rear flank. Mice were then allocated into four groups (5 mice in each group), including control, leptin (1 mg/kg), leptin (1 mg/kg) plus 3-MA (1 mg/kg), and 3-MA (1 mg/kg) alone. The mice were treated with leptin and 3-MA by intraperitoneal injection every 36 h for 4 weeks. (A) Representative images of mice bearing tumors from each group at the end of the treatment were shown. (B) After 4-week period of treatment, tumor tissues were excised and representative images for each group were presented. (C) Tumor growth rate was monitored during treatment period by measuring tumor volume as described in Materials and methods. (D) Tumor tissues were collected at the end of treatment period and their weights were immediately measured. (E) Total cellular extracts were prepared from tumor tissues and protein expression levels of LC3I/II were determined by Western blot analysis. Representative images for three samples from each group were presented.
