# Supplementary material for: Autophagy activation and SREBP‐1 induction contribute to fatty acid metabolic reprogramming by leptin in breast cancer cells
Source: Mol Oncol. 2020 Dec 5;15(2):657–78. doi: 10.1002/1878-0261.12860 (PMC7858107; doi:10.1002/1878-0261.12860)
Supplement: Supplementary file 4 — Fig. S4. The effect of leptin on cell viability of MDA‐MB‐231 breast cancer cells. [file MOL2-15-657-s004.pdf]

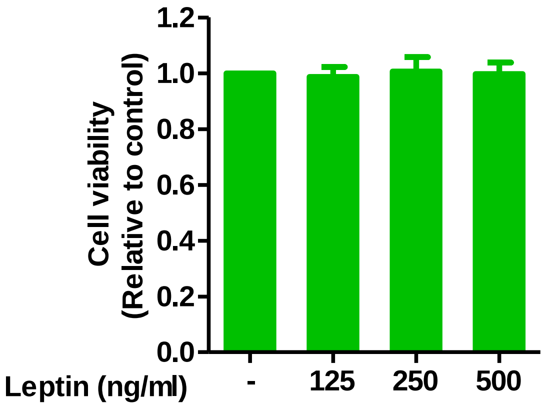

Fig. S4. *The effect of leptin on cell viability of MDA-MB-231 breast cancer cells*. MDA-MB-231 cells were treated with different concentrations of leptin for 48 h followed by MTS assay as described in Methods.
