# Supplementary material for: Autophagy activation and SREBP‐1 induction contribute to fatty acid metabolic reprogramming by leptin in breast cancer cells
Source: Mol Oncol. 2020 Dec 5;15(2):657–78. doi: 10.1002/1878-0261.12860 (PMC7858107; doi:10.1002/1878-0261.12860)
Supplement: Supplementary file 5 — Table S1. Sequences of siRNA duplexes and PCR primers used in the study. [file MOL2-15-657-s005.docx]

**Table S1.** Sequences of siRNA duplexes and PCR primers used in the study

| **Target gene** | **Primer** | **Nucleotide** |
| --- | --- | --- |
| LC3B siRNA | F | 5’- GACUGUCUCGUUUAGACUG-3’ |
|  | R | 5’- CAGUCUAAACGAGACAGUC-3’ |
| SREBP1 siRNA | F | 5’- CCACCGUUUCUUCGUGGAU-3’ |
|  | R | 5’- AUCCACGAAGAAACGGUGG-3’ |
| FASN siRNA#1 | F | 5’- UCAACCUGGACAGCUCACU-3’ |
|  | R | 5’-AGUGAGCUGUCCAGGUUGA-3’ |
| FASN siRNA#2 | F | 5’-CUCAACUUCCGCGACAUCA-3’ |
|  | R | 5’-UGAUGUCGCGGAAGUUGAG-3’ |
| SREBP-1 primer | F | 5’-GCAAGGCCATCGACTACATT-3’ |
|  | R | 5’-GGTCAGTGTGTCCTCCACCT-3’ |
| FASN primer | F | 5’-AAGGACCTGTCTAGGTTTGATGC-3’ |
|  | R | 5’-TGGCTTCATAGGTGACTTCCA-3’ |
| ACC_α_ primer | F | 5’-ATGTCTGGCTTGCACCTAGTA-3’ |
|  | R | 5’-CCCCAAAGCGAGTAACAAATTCT-3’ |
| FADS1 primer | F | 5’-ACAGTGACAAAAGGCTCGGA-3’ |
|  | R | 5’-ACTTGACCAGAGGCAGCTTT-3’ |
| FADS2 primer | F | 5’-TTGTGTGTGCGTGTTGTTGG-3’ |
|  | R | 5’-ACCAATCAGCAGGGGTTTCA-3’ |
| ACLY primer | F | 5’-TGCAAAGTGAAGTGGGGTGA-3’ |
|  | R | 5’-TTTGGGGTTCAGCAAGGTCA-3’ |
| SCD-1 primer | F | 5’-AAAGCGAGGTGGCCATGTTA-3’ |
|  | R | 5’-TCATGCCTCAAAACTGCCCT-3’ |
| SCD-5 primer | F | 5’-TGGTGCTCATGTGCTTTGTG-3’ |
|  | R | 5’-AGGGCTGATGTGCTTGTCAT-3 |
| GADPH primer | F | 5’-ACCACAGTCCATGCCATCAC-3’ |
|  | R | 5’-TCCACCACCCTGTTGCTGTA-3’ |
